# Throughfall Reduction Other than Nitrogen Addition Threatens Soil Multifunctionality in a Subtropical Moso Bamboo Forest

**DOI:** 10.3390/microorganisms14081619

**Published:** 2026-07-24

**Authors:** Shiquan Su, Xiao Xiong, Yanqi Han, Yaping Di, Yanli Jing, Yi Wang

**Affiliations:** 1 Key Laboratory of Bamboo and Rattan Science and Technology of State Forestry and Grassland Administration, Institute of Resources and Environment, International Centre for Bamboo and Rattan, Beijing 100102, China; sushiquan0116@163.com (S.S.); 17262247641@163.com (X.X.); 2 Guangxi Key Laboratory of Forest Ecology and Conservation, College of Forestry, Guangxi University, Nanning 530004, China; 13253092859@163.com; 3 Ecological Conservation, Restoration and Resource Utilization in Forest and Wetland Key Laboratory of Sichuan Province, Sichuan Academy of Forestry, Chengdu 610081, China; dyp7200@163.com; 4 School of Life Sciences (School of Ecological Forestry), Mianyang Normal University, Mianyang 621000, China; jingyanli@126.com; 5 Sichuan Jiuzhai Valley National Ecological Quality Comprehensive Monitoring Station, Mianyang Normal University, Mianyang 621000, China

**Keywords:** soil multifunctionality, throughfall reduction, nitrogen addition, microbial community structure, moso bamboo

## Abstract

Understanding the relationship between biodiversity and ecosystem multifunctionality remains a central challenge in ecology. Although biodiversity is generally associated with enhanced multifunctionality, this relationship can vary across ecosystems and environmental contexts. It remains unclear how concurrent global changes, such as drought and nitrogen deposition, modulate biodiversity–multifunctionality relationships, in particular the moso bamboo forests in subtropical China. We conducted a field manipulation experiment combining throughfall reduction and nitrogen addition to simulate drought and nitrogen deposition, and applied amplicon sequencing to examine how these global change factors independently and interactively influence soil microbial diversity, multifunctionality, and their interrelationships. We found that throughfall reduction significantly reduced soil multifunctionality, likely due to lower soil water availability, and nitrogen addition exacerbated this negative effect. However, environmental changes did not affect bacterial and fungal α-diversity, nor was soil multifunctionality correlated with microbial diversity. Instead, shifts in soil multifunctionality were closely linked to changes in microbial community composition and co-occurrence network structure. In particular, increasing relative abundances of Acidobacteriota, Myxococcota, and Chytridiomycota were positively associated with higher soil multifunctionality. Our findings demonstrate that soil multifunctionality responses to throughfall reduction and nitrogen addition are primarily mediated through microbial community reorganization rather than diversity loss, providing new insights into the mechanisms linking global change drivers, microbial communities, and ecosystem functioning.

## 1. Introduction

Ecosystem multifunctionality refers to the capacity of an ecosystem to simultaneously maintain multiple ecological functions and services, such as primary productivity, nutrient provisioning and cycling [1,2]. When these functions are primarily soil-related, the term soil multifunctionality is widely adopted [3,4]. Soil multifunctionality is governed by a suite of biotic and abiotic factors, including biodiversity, species abundance, and environmental conditions such as soil nutrients and soil water content [2,5]. Among these, biodiversity is often considered a key driver sustaining multifunctionality, with numerous studies showing that greater biodiversity tends to enhance multifunctionality across spatial scales [6]. However, under local conditions, species interactions along nutrient and water availability gradients may lead to reduced biodiversity under high-functioning scenarios [7,8,9], highlighting that our current understanding of biodiversity–multifunctionality relationships remains incomplete and potentially context-dependent on spatial scale, climatic variability, and edaphic conditions. Moreover, although microbial diversity has been frequently used to explain variations in soil multifunctionality, increasing evidence suggests that specific microbial taxa and their interactions also play key roles in regulating ecosystem functions [10]. Consequently, compared with microbial diversity alone, microbial community composition and co-occurrence network complexity may provide stronger predictors of soil multifunctionality.

Soil microbes represent a substantial component of biodiversity and are integral to multiple ecosystem processes [11]. However, a sole focus on microbial diversity effects may ignore the substantial influence of species identity and dominance, which have been shown to explain a larger share of variation in multifunctionality than biodiversity per se [12,13]. While biodiversity contributes to functional redundancy, dominant species can exert direct effects on ecosystem processes, as well as indirect effects by altering diversity or abundances of other biota [14,15]. Changes in relative abundances of key microbial functional groups, other than alpha diversity, promote soil multifunctionality [8]. Additionally, bacteria can affect diversity and community compositions of soil fungi communities, through competition, inhibition, and mutualism, forming intricate co-occurrence networks [16], whose structural complexity and stability have been positively associated with multifunctionality [17]. Although previous studies have largely focused on bacterial or fungal networks separately [18], cross-domain interactions among bacteria and fungi are common in natural soils and may be crucial to ecosystem function [5]. Nevertheless, whether more complex cross-domain microbial networks are associated with greater multifunctionality in ecosystems with changing environments, e.g., throughfall reduction and nitrogen addition, remains largely unexplored, as does the relative importance of bacterial versus fungal communities in sustaining multifunctionality.

Global change remains the central issue influencing ecosystem services and functions. Changes in precipitation and nitrogen deposition exerted a significant influence on soil microbial communities and multifunctionality [19]. For example, nitrogen addition alters soil bacterial communities and improves soil multifunctionality in temperate forests [20]. However, it has also been reported to weak biodiversity–multifunctionality relationships in a temperate grasslands [21]. In the bamboo forests of subtropical China, nitrogen addition inhibited microbial extracellular enzyme activities relating to C turnover, while promoted those relating to nitrogen acquisition [22]. Taking all this together, the effects of nitrogen addition on soil microbial communities and soil functions seemed to be complicated. In contrast, throughfall reduction generally reduced soil microbial diversity and multifunctionality [10,23,24], regardless of vegetation and soil types, potentially due to reduced soil water availability for microbial metabolism. Nevertheless, the conjunction effects of throughfall reduction and nitrogen addition on soil multifunctionality and microbial communities remain elusive, necessitating further investigation.

The moso bamboo (*Phyllostachys edulis*) is widely distributed in southern China, with a total forest area of 5.28 × 10^6^ ha in 2021, accounting for about 70% of the total bamboo forest area in China [25]. The Sichuan Basin, which lies in the subtropical regions of western China, is suffering from reduced precipitation and increasing nitrogen deposition [26,27], making it an ideal base in testing the above research gaps. Thereby, in this study, we conducted a field manipulation experiment incorporating throughfall reduction and nitrogen addition (for more information, see Section 2.2) to investigate the effects of simulated drought and nitrogen deposition on soil multifunctionality and soil microbial communities. Our previous studies have revealed that our study site was a N-enriched bamboo forest ecosystem, and the effect of drought on soil C and N cycling depends on soil nitrogen availability, which was attributed to variation in microorganisms [28]. Furthermore, the positive response of moso bamboo transpiration to nitrogen addition was detected, which enhances soil water uptake by plants, and thus further accelerated the restriction of soil water content on microorganisms [29]. Specifically, we hypothesized that (i) throughfall reduction inhibits soil multifunctionality by reducing the soil water content, and nitrogen addition can further exacerbate the inhibitory effect of throughfall reduction on soil multifunctionality. (ii) Throughfall reduction and nitrogen addition may not significantly influence soil microbial diversity but community composition, with key taxa relating to soil multifunctionality being inhibited by throughfall reduction and aggravated by nitrogen addition. (iii) Relative abundances of key oligotrophic taxa such as Acidobacteriota, other than soil microbial alpha diversity, would be the primary driver of multifunctionality under the co-occurrence scenarios of throughfall reduction and nitrogen addition.

## 2. Materials and Methods

### 2.1. Site Description

This study was conducted at the Changning Bamboo Forest Ecosystem Research Station (28°27′59.4″ N, 105°01′6.4″ E). This region lies in the transitional zone between the Yunnan–Guizhou Plateau and the Sichuan Basin, at the junction of the southwestern clumping bamboo distribution area and the range of mixed Jiangnan bamboo and alpine bamboo in Southwest China. The mean annual precipitation and the mean annual temperature are about 1141.7 mm and 18.3 °C, respectively. The soil in the study site was Cambisols (FAO classification). The main vegetation includes bamboo forests dominated by *Phyllostachys edulis*, *Neosinocalamus affinis*, *Bambusa rigida*, and *Pleioblastus amarus*, as well as stands of *Quercus* spp. and *Cupressus* spp.

### 2.2. Experimental Design

The experiment was initiated in April 2017 for throughfall reduction and in June 2017 for nitrogen addition at the research station, respectively, within a *Phyllostachys edulis* plantation [28,29]. A randomized block design with four blocks at least 100 m apart was adopted [28], including four treatments with four replicates each: control (CK, no nitrogen addition and no throughfall reduction treatment), throughfall reduction (Tr, covering about 50% area for throughfall exclusion), nitrogen addition (Na, 10 g nitrogen m^−2^ yr^−1^), and combined throughfall reduction and nitrogen addition (Tr + Na, covering about 50% area for throughfall exclusion plus 10 g nitrogen m^−2^ yr^−1^).

Briefly, stainless-steel-frame structures were installed 2–2.5 m above the ground beneath the canopy for the Tr treatment, then transparent polyethylene sheets were arranged to covering 50% of the plot area, considering the spatial distribution of bamboo clumps. Three gutters were installed in each plot to collect and divert the intercepted water outside the plots. For Na treatment, ammonium nitrate (NH_4_NO_3_) solution was applied beneath the canopy every two months at the designated rate, while CK and Tr plots received the same volume of deionized water. The details of plot information were demonstrated by [28,30].

### 2.3. Soil Sampling

For each plot, 5 subsamples were randomly collected with a soil auger (5 cm in diameter) from 0 to 10 cm depth in August 2020, and the subsamples were mixed to produce a representative composite sample. After sampling, all the soil subsamples were sieved through a 2 mm mesh and divided into three portions for further analysis immediately: one was stored at 4 °C for enzyme activity measurements, one was frozen at −20 °C for the analysis of microbial communities, and the other was left to air dry for the assessment of the soil properties.

### 2.4. Measuring Soil Properties and Extracellular Enzyme Activities

Soil organic carbon (SOC) and total nitrogen (TN) concentrations were analyzed using an Elemental Analyzer (ECS 4010 CHNSO, Costech Analytical Tecnologies Inc., Valencia, CA, USA). Total phosphorus (TP) and available P (AP) were measured by using a Smartchem Discrete Auto Analyzer (Smartchem 300, Westco Scientific Instruments, Rome, Italy). Soil pH was determined with a pH meter (PHS-3C, INESA Scientific Instrument Co., Ltd., Shanghai, China) in a soil-distilled water 1:2.5 (*w*/*v*) water suspension. Ammonium nitrogen (NH_4_^+^-N) and nitrate nitrogen (NO_3_^−^-N) were obtained by measuring 2 mol L^−1^ KCl extracts using a Smartchem Discrete Auto Analyzer (Smartchem 300, Westco Scientific Instruments, Rome, Italy).

Extracellular enzyme activities involving in the carbon (α-glucosidase [AG: 4-Methylumbelliferyl α-D-glucopyranoside], β-glucosidase [BG: 4-Methylumbelliferyl β-D-glucopyranoside], cellobiohydrolase [CB: 4-Methylumbelliferyl β-D-cellobioside], β-xylosidase [BX: 4-Methylumbelliferyl-β-D-xylopyranoside]), nitrogen (β-1,4-N-acetylglucosaminidase [NAG: 4-Methylumbelliferyl N-acetyl-β-D-glucosaminide], leucine aminopeptidase [LAP: L-Leucine-7-amido-4-methylcoumarin hydrochloride] (Sigma-Aldrich, St. Louis, MO, USA)), and phosphorus (acid phosphatase [ACP: 4-Methylumbelliferyl phosphate]) cycles were measured using a microplate fluorometric multi-substrate assay as outlined by German [31]. Two oxidative enzymes (polyphenol oxidase [PPO] and phenoloxidase peroxidase [PHE]) involved in lignin degradation were determined using L-3, 4-dihydroxyphenylalanine (DOPA: L-dihydroxyphenylalanine) as the substrate. All the reagents were obtained from Sigma-Aldrich (Sigma-Aldrich, St. Louis, MO, USA). The extracellular enzyme activities were read on a PerkinElmer LAMBDA 35 plate reader (PerkinElmer, Waltham, MA, USA, excitation filter at 355 nm and emission filter at 460 nm).

### 2.5. Biodiversity Analysis

High-throughput amplicon sequencing was used to estimate bacterial and fungal diversity. Microbial DNA was extracted using the FastDNA^®^ SPIN Kit for Soil (produced by MP Biomedicals, Irvine, CA, USA). Then, the V3–V4 hypervariable regions of the bacterial 16S rRNA gene were amplified using a PCR Amplifier (2720, ABI, Foster City, MA, USA), with the primer set 515F (5′-ACTCCTACGGGAGGCAGCAG-3′) and 806R (5′-GGACTACHVGGGTWTCTAAT-3′), while the ITS1 region of fungal sequences was amplified using the primer set ITS1F (5′-CTTGGTCATTTAGAGGAAGTAA-3′) and ITS2R (5′-GCTGCGTTCTTCATC GATGC-3′).

The 25 μL reaction mixtures for the amplification of both 16S rRNA and ITS genes were identical, including 1 μL of each primer (10 μM, 515F and ITS1F primers were linked with a unique 12 bp barcode for each sample), 2 μL of DNA template (10 ng μL^−1^), 8.5 μL of ddH_2_O, and 12.5 μL of MasterMix containing Taq DNA Polymerase, PCR Buffer, Mg^2+^ and dNTPs (CWBIO, Beijing, China). Amplification programs were as follows: (i) for the 16S rRNA gene, initial denaturing at 95 °C for 3 min, followed by 30 cycles of 95 °C for 30 s, 55 °C for 30 s, and 72 °C for 30 s, and a final extension at 72 °C for 10 min; and (ii) for the ITS gene, initial denaturing at 95 °C for 3 min, and 27 cycles of 95 °C for 30 s, 55 °C for 30 s, and 72 °C for 30 s, then a final extension at 72 °C for 10 min. The amplicon sizes were determined and selected by running 2% agarose gel electrophoresis in 1.0× TAE buffer, and the amplicons were purified using the AxyPrep DNA Gel Extraction Kit (AP-GX-250, Axygen, Union City, CA, USA). Samples were normalized in equimolar amounts in the final mixture. DNA library construction was performed on an Illumina platform following the manufacturer’s instructions. Finally, target DNA was sequenced on an Illumina Novaseq 6000 platform (Illumina, San Diego, CA, USA).

FLASH v1.2.11 (Magoč and Salzberg, 2011) was used to merge paired-end reads [32]. Sequences with length < 300 bp for 16S sequences or with length < 200 bp for ITS sequences, more than 2 ambiguous base ‘N’ and the average base quality score < 30 were removed. Then, the QIIME2 pipeline was used for downstream analyses [33]. The amplicon sequence variants (ASVs) were obtained using the DADA2 algorithm. All samples were resampled to 11,500 sequences and 24,000 per sample, respectively, for bacteria and fungi, referring to the sample with the lowest sequence reads. To assign taxonomic affiliations, the SILVA ribosomal RNA gene database (Release 138) [34] and UNITE database (v10.0) [34] were used respectively for the 16S rRNA and ITS genes.

Raw sequence data have been deposited in the National Genomics Data Center (https://ngdc.cncb.ac.cn/) under BioProject accession number CNP0009868. The taxonomic diversity of bacteria and fungi was represented by the richness (observed ASVs), while the faith’s phylogenetic diversity (faith’s PD) was used to represent their phylogenetic diversity. These diversity indices were calculated using the *microeco* R package (version 4.4.3) [35].

### 2.6. Measuring Soil Multifunctionality

To quantify soil multifunctionality under the throughfall reduction and nitrogen addition treatments, we assessed 15 key soil functions that reflect both fast (process-based) and slow (resource pool-based) soil processes. The process-based functions were mainly represented by the extracellular enzyme activities involving in the C, N and P cycling: (1) AG, (2) BG, (3) CB, (4) BX, (5) PHE, (6) PPO, (7) NAG, (8) LAP, (9) ACP, and the nutrient pool based functions: (10) SOC, (11) TN, (12) TP, (13) TAP, and soil available N, represented by (14) NH_4_^+^-N and (15) NO_3_^−^-N contents. More specifically, PPO, PHE, AG, CB, BX and BG are good proxies of process rates involved in soil organic matter decomposition; NAG and LAP are key processes involving nitrogen supplying; and ACP refers to soil phosphorus cycling processes. These functions are good indicators of biotic processes measured as rates, representing effects of biological processes on a shorter time scale [36]. Moreover, the nutrient pool-based functions are good proxies of energy stocking or matters representing effects of biological processes on a longer time scale [36].

To avoid overweighting highly correlated functions in the multifunctionality index, using the above fifteen functional parameters, a weighted multifunctionality index was calculated to down-weight highly correlated functions following three main procedures. Firstly, all functions were standardized by dividing their maximum observed value. Then, following the procedures proposed by Manning et al. [2], a clustering analysis was conducted to obtain the weight coefficient of each function. The optimal number of clusters (k) was determined using two complementary criteria: (1) the elbow method based on the within-cluster sum of squares (WSS), where the inflection point indicates the most parsimonious k; and (2) the average silhouette width, which measures cluster separation quality (values > 0.7 indicate strong cluster structure). The WSS declined sharply from k = 2 (0.599) to k = 3 (0.034), after which additional clusters contributed negligible variance reduction. Despite the silhouette width at k = 2 showing the highest value (0.925), the width value at k = 3 was well within the “excellent” range (0.820, which is greater than >0.7). Together, these two criteria converged on k = 3 as the most parsimonious and biologically interpretable choice (Figure S1). Each cluster was assigned 1 as the weight coefficient, and the parameter weight coefficient was 1, divided by the number of functions in each cluster. Finally, the weighted soil multifunctionality index was calculated with the following formula [1,2]:

(1) $$SMF=\frac{1}{m}\times \sum_{i=1}^{n}a_{i}{\times f}_{i}$$

where *m* is the number of function clusters; *n* represents the number of functions selected; and *a_i_* and *f_i_* represent the weight coefficient and standardized values of function *i*, respectively.

### 2.7. Statistical Analyses

All statistical analyses were conducted using R version 4.4.3 [37]. Differences in soil multifunctionality among drying and N addition treatments were assessed using the non-parametric Friedman test via the “friedman” function of the “agricolae” R package [38]. Relationships between soil multifunctionality and microbial diversity indices were evaluated using linear mixed-effects models, with Block included as a random intercept to account for the randomized block design. The proportion of variance explained by each fixed effect (Marginal R^2^) and by the full model including the random effect (Conditional R^2^) was calculated. Where the random effect variance was estimated at zero, only Marginal R^2^ is reported. To explore potential interactions between bacteria and fungi, cross-domain co-occurrence networks were constructed. Spearman’s correlation-based co-occurrence networks were constructed to infer the potential biotic interactions among microbial communities. To minimize potential spurious correlations from the rare taxa [17,39], we focused on the dominant taxa, which are widely distributed and have strong functional importance [17]. Specifically, for each microbial group, we kept those ASVs with relative abundance higher than 0.01%. The ASV tables of microbial groups were then merged, and correlations between any ASV with a robust Spearman’s rho (i.e., ≥0.65) and a false discovery rate < 0.001 were used to construct networks. This cutoff threshold has been widely used in the literature and thus is comparable among studies [17]. In addition to that, it has a meaning beyond mathematics, i.e., it reveals the organisms that are strongly correlated (possibly, strongly interact) with each other [17,40]. The topological properties, including number of nodes and edges, average degree, average path length, clustering coefficient, ratio of positive/negative links and connectivity, were calculated. The average degree indicates the average connections of each node with another unique node in a network; average path length refers to the average distance between any pair of nodes; and the clustering coefficient describes the extent to which nodes are clustering together [41,42]. In general, greater values of node and edge numbers, average degree, and clustering coefficient and smaller values of average path length indicate that networks are more connected, presenting more complex potential interactions among microbes [43]. Network was constructed using the “igraph” package [44], and visualized using the interactive Gephi software (https://gephi.org). Additionally, to gain the topological properties of each sample, we extracted the subnetworks by preserving the ASVs of individual soil samples using the ‘induced_subgraph’ function in the “igraph” package.

Network topological features, including the number of nodes and edges, average degree, average path length, clustering coefficient, positive-to-negative link ratio, and overall connectivity, were calculated and used to examine their associations with soil multifunctionality. Differences in alpha diversity and network properties across plant families were also tested using the Kruskal–Wallis test. Differences in beta diversity (community compositions) of soil bacterial and fungal communities were tested using permutational multivariate analysis of variance (PERMANOVA) and visualized using non-metric multidimensional scaling (NMDS) ordinations.

Mantel tests were conducted to evaluate correlations between bacterial and fungal β-diversity, network properties, and soil multifunctionality. To further identify dominant microbial taxa associated with multifunctionality, we applied random forest regression models based on the relative abundances of dominant bacterial and fungal phyla. The optimal number of variables per split (mtry = 20) was determined by minimizing out-of-bag (OOB) mean squared error via tuneRF. The final model was run with 9999 trees to ensure robust convergence. Model performance was assessed using OOB R^2^ and percent variance explained (Figure S2). OOB error stabilization was visualized by plotting the mean squared error (MSE against tree number with a 50-tree rolling mean) to assess whether the number of trees was sufficient. Predictor importance was assessed by permuting each variable and calculating the percentage increase in MSE; a higher increase in MSE indicates a stronger contribution to the model. Random forest analyses were conducted using the “rfPermute” R package [45].

## 3. Results

### 3.1. Effects of Throughfall Reduction and Nitrogen Addition on Soil Properties and Soil Multifunctionality

**Figure 1:**
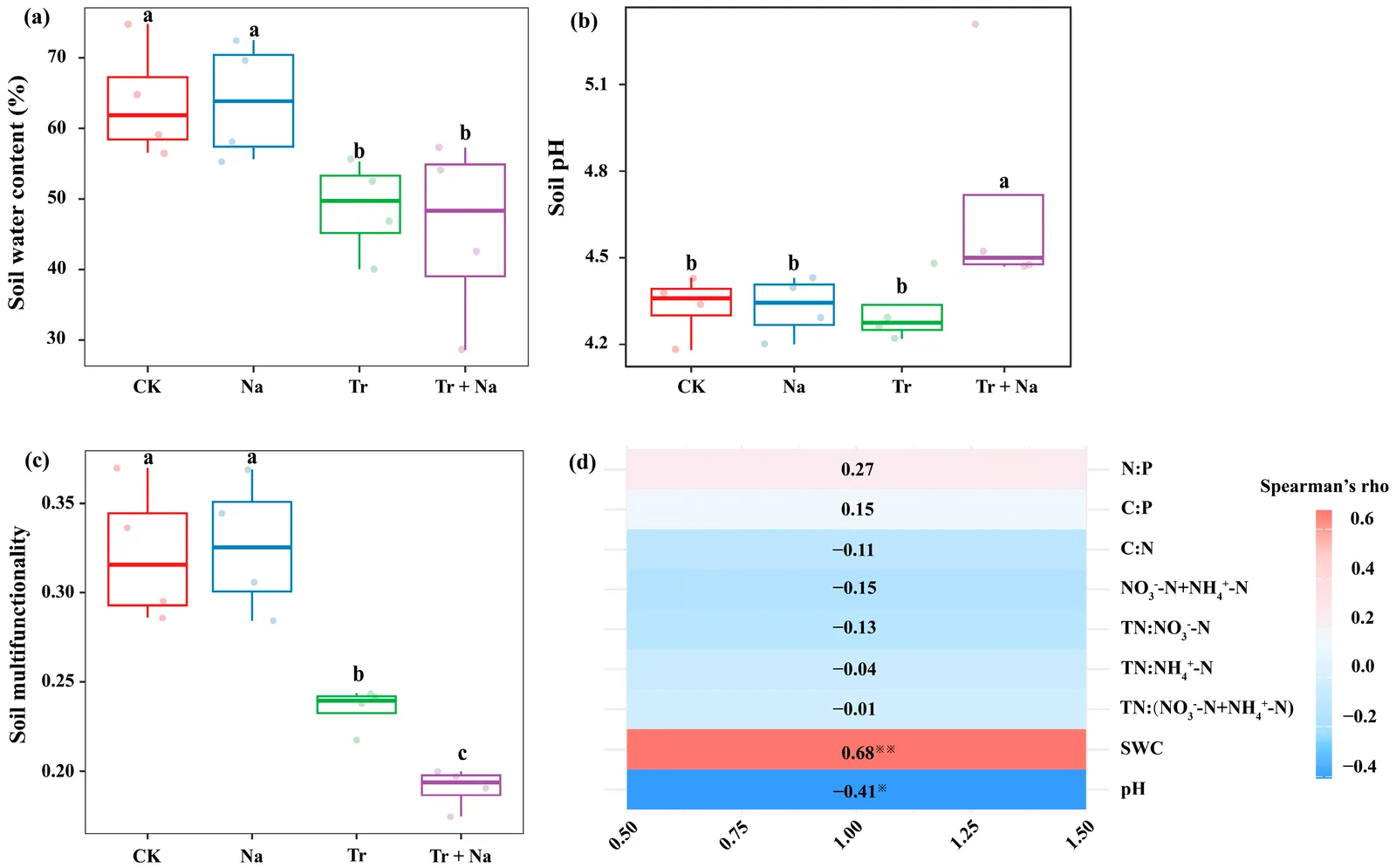
(**a**,**b**) Variation in soil properties (mean values) among treatments. (**c**) Variation in soil multifunctionality (mean values) among treatments. (**d**) Spearman’s correlation coefficients (ρ) between soil multifunctionality and soil properties. In panel (**a**–**c**), different lowercase letters denote significant differences among treatments based on Friedman tests with block as the blocking factor (*χ*^2^ = 9.6 (SWC), 7.46 (pH) and 10.8 (SMF), *p* < 0.05; post hoc LSD test, α = 0.05). In panel (**d**), asterisks indicate false discovery rate (FDR)-adjusted significance: * *p* < 0.05, ** *p* < 0.01. Treatment abbreviations: CK, control (no nitrogen addition and no throughfall reduction treatment), Tr, throughfall reduction (covering about 50% area for throughfall exclusion), Na, nitrogen addition (10 g nitrogen m^−2^ yr^−1^), and Tr + Na, combined throughfall reduction and nitrogen addition (covering about 50% area for throughfall exclusion plus 10 g nitrogen m^−2^ yr^−1^).

Across treatments, soil properties and soil multifunctionality varied significantly (*p* < 0.05), with the highest values of soil water content and soil multifunctionality observed in the CK and Na treatments, followed by lower values in the Tr treatment and Tr + Na treatment (Figure 1a,c). Conversely, there was the highest value of soil pH in the Tr + Na treatment (Figure 1b). Further analysis indicated that treatment-induced changes in soil water content and soil pH were the primary abiotic factors driving variation in soil multifunctionality (Figure 1d).

### 3.2. Effects of Throughfall Reduction and Nitrogen Addition on Soil Bacterial and Fungal Communities

**Figure 2:**
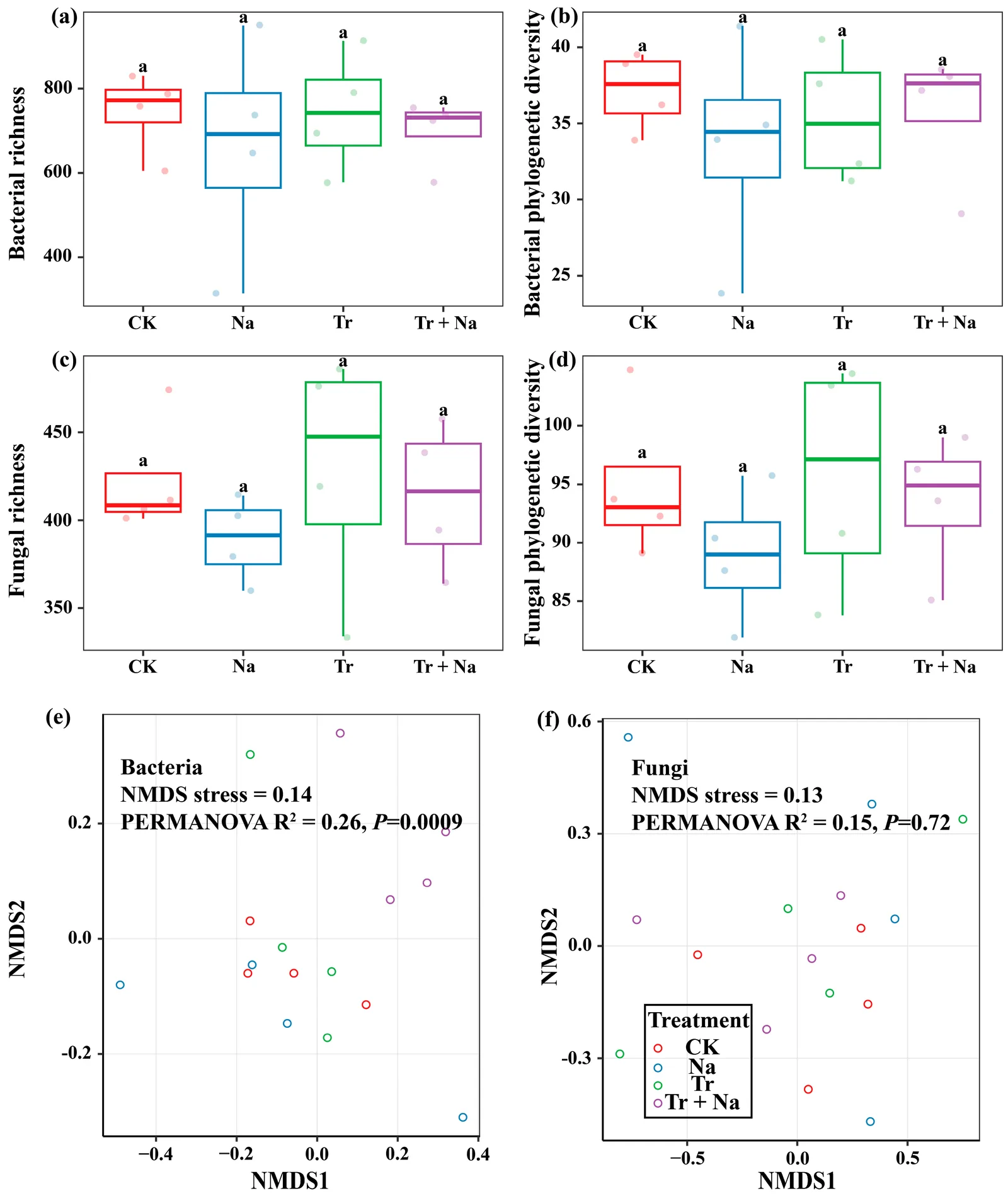
(**a**–**d**) Boxplots showing the distribution of alpha diversity indices of soil bacterial and fungal communities among treatments. In panel (**a**–**d**), different lowercase letters denote significant differences among treatments based on Friedman tests with block as the blocking factor (*χ*^2^ = 1.77, 0.96, 0.55, 0.68 respectively, *p* > 0.05). (**e**,**f**) Non-metric multidimensional scaling (NMDS) ordinations coupled with permutational multivariate analysis of variance (PERMANOVA) illustrating differences in community composition. Richness and phylogenetic diversity are represented by observed ASVs and Faith’s PD, respectively. Treatment abbreviations: CK, control (no nitrogen addition and no throughfall reduction treatment), Tr, throughfall reduction (covering about 50% area for throughfall exclusion), Na, nitrogen addition (10 g nitrogen m^−2^ yr^−1^), and Tr + Na, combined throughfall reduction and nitrogen addition (covering about 50% area for throughfall exclusion plus 10 g nitrogen m^−2^ yr^−1^).

**Figure 3:**
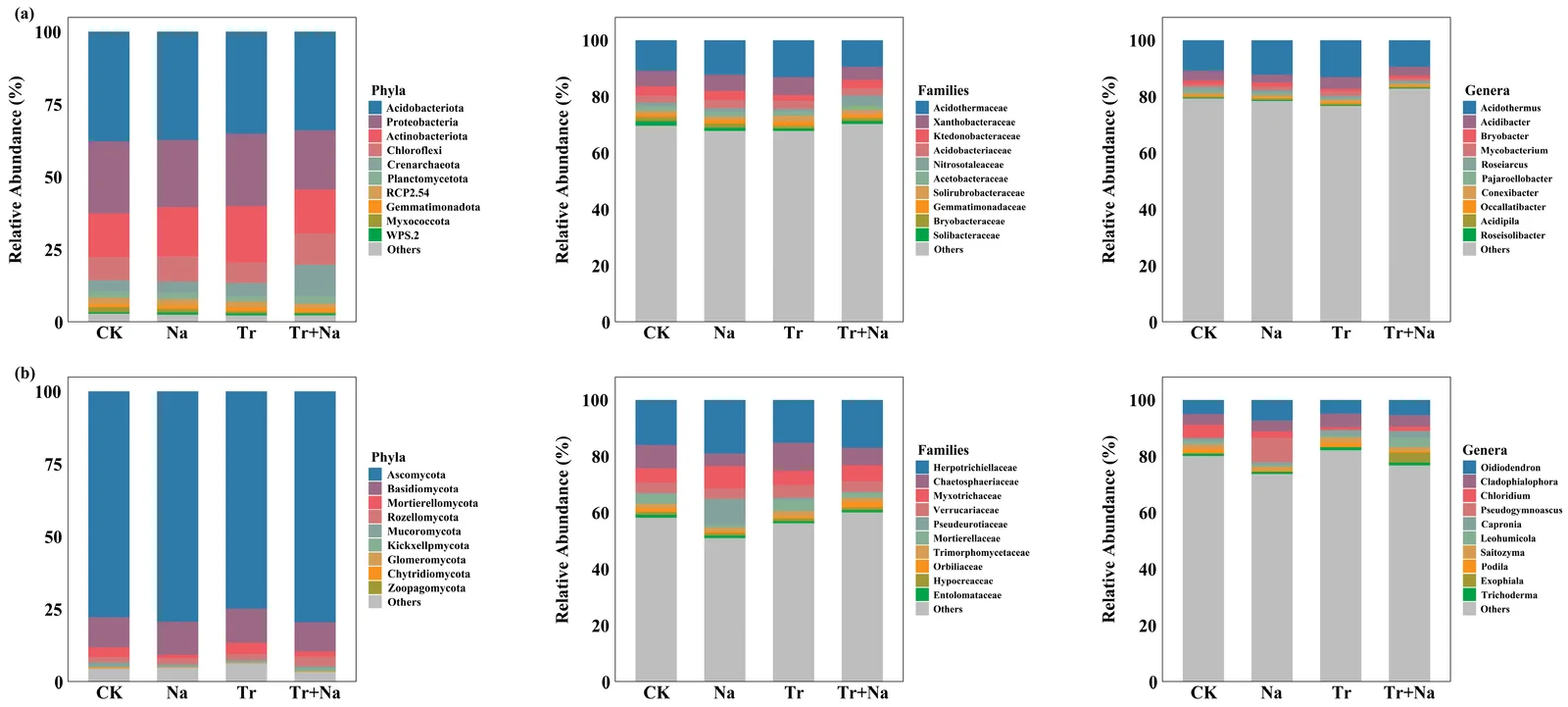
Bar plots showing variations in relative abundances of dominant bacterial (**a**) and fungal (**b**) taxa (top 10 phyla, families and genera) among treatments. Treatment abbreviations: CK, control (no nitrogen addition and no throughfall reduction treatment), Tr, throughfall reduction (covering about 50% area for throughfall exclusion), Na, nitrogen addition (10 g nitrogen m^−2^ yr^−1^), and Tr + Na, combined throughfall reduction and nitrogen addition (covering about 50% area for throughfall exclusion plus 10 g nitrogen m^−2^ yr^−1^).

Given their central roles in soil biogeochemical cycling, bacterial and fungal communities—as well as their potential inter-domain interactions—were examined as potential biotic drivers of soil multifunctionality under throughfall reduction and nitrogen addition. Alpha diversity indices of bacterial and fungal communities did not differ significantly among treatments, although slight shifts were observed (Figure 2a). In contrast, beta diversity analyses revealed that Tr + Na treatment significantly affected bacterial community composition (PERMANOVA, *p* = 0.009), but not fungal communities (*p* > 0.05) (Figure 2b). In the bacterial NMDS ordination, Tr + Na treatments were clearly separated from the others, whereas no distinct clustering was observed for fungal communities (Figure 2b). Specifically, bacterial communities were dominated by Acidobacteriota (36.07% in relative abundances), Proteobacteria (23.32%), Actinobacteriota (16.57%), and Chloroflexi (8.70%), with Acidothermaceae (11.40%), Xanthobacteraceae (5.57%), and Ktedonobacteraceae (2.90%) as the predominant families and Acidothermus (11.40%), Acidibacter (3.46%), and Bryobacter (1.20%) as the dominant genera. Likewise, fungal communities were dominated by Ascomycota (77.87%), Basidiomycota (10.89%), and Mortierellomycota (2.60%), with Herpotrichiellaceae (16.83%), Chaetosphaeriaceae (7.22%), and Myxotrichaceae (5.90%) representing the major families and Oidiodendron (5.69%), Cladophialophora (4.14%), and Chloridium (2.34%) the dominant genera. Notably Myxococcota showed significantly (Kruskal–Wallis *p* < 0.05) higher and lower relative abundances in the CK and Tr + Na treatment, respectively, similar to the observed decline in soil multifunctionality (Figure 3). The inter-domain co-occurrence network comprised both bacterial and fungal nodes, with several nodes exhibiting high connectivity (Figure S3a). Additionally, the key taxa in the networks are mostly positively correlated, indicating mutualistic relationships.

### 3.3. Relationships Between Soil Multifunctionality and Bacterial and Fungal Communities Under Throughfall Reduction and Nitrogen Addition

**Figure 4:**
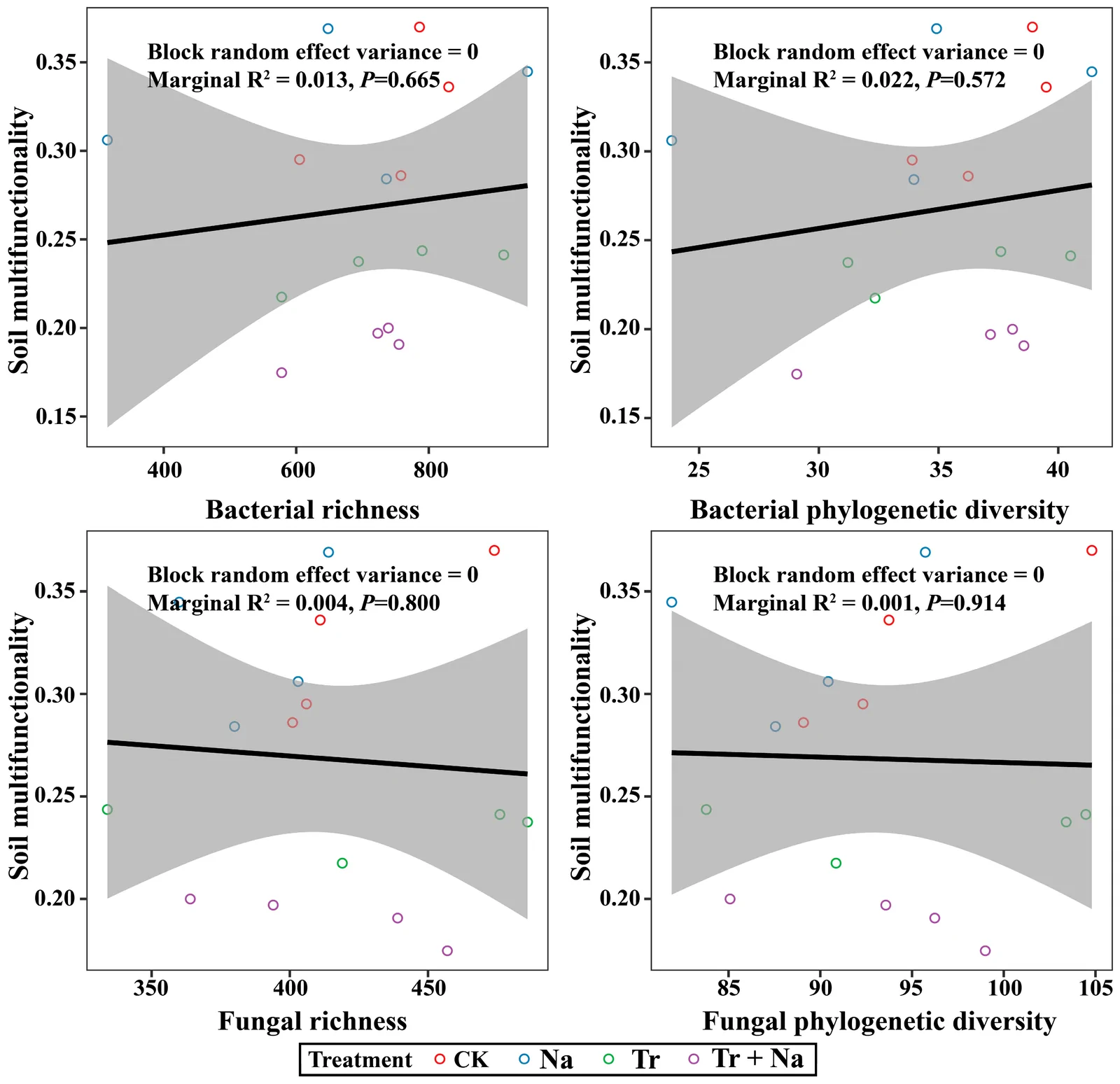
Scatterplots showing non-significant relationships between soil multifunctionality and microbial diversity indices, including richness (observed ASVs) and Faith’s phylogenetic diversity of soil bacterial and fungal communities. Lines represent linear mixed-effects model fits with Block as a random intercept; shaded areas indicate 95% confidence intervals. None of the relationships were significant (all *p* > 0.05). Treatment abbreviations: CK, control (no nitrogen addition and no throughfall reduction treatment), Tr, throughfall reduction (covering about 50% area for throughfall exclusion), Na, nitrogen addition (10 g nitrogen m^−2^ yr^−1^), and Tr + Na, combined throughfall reduction and nitrogen addition (covering about 50% area for throughfall exclusion plus 10 g nitrogen m^−2^ yr^−1^).

**Figure 5:**
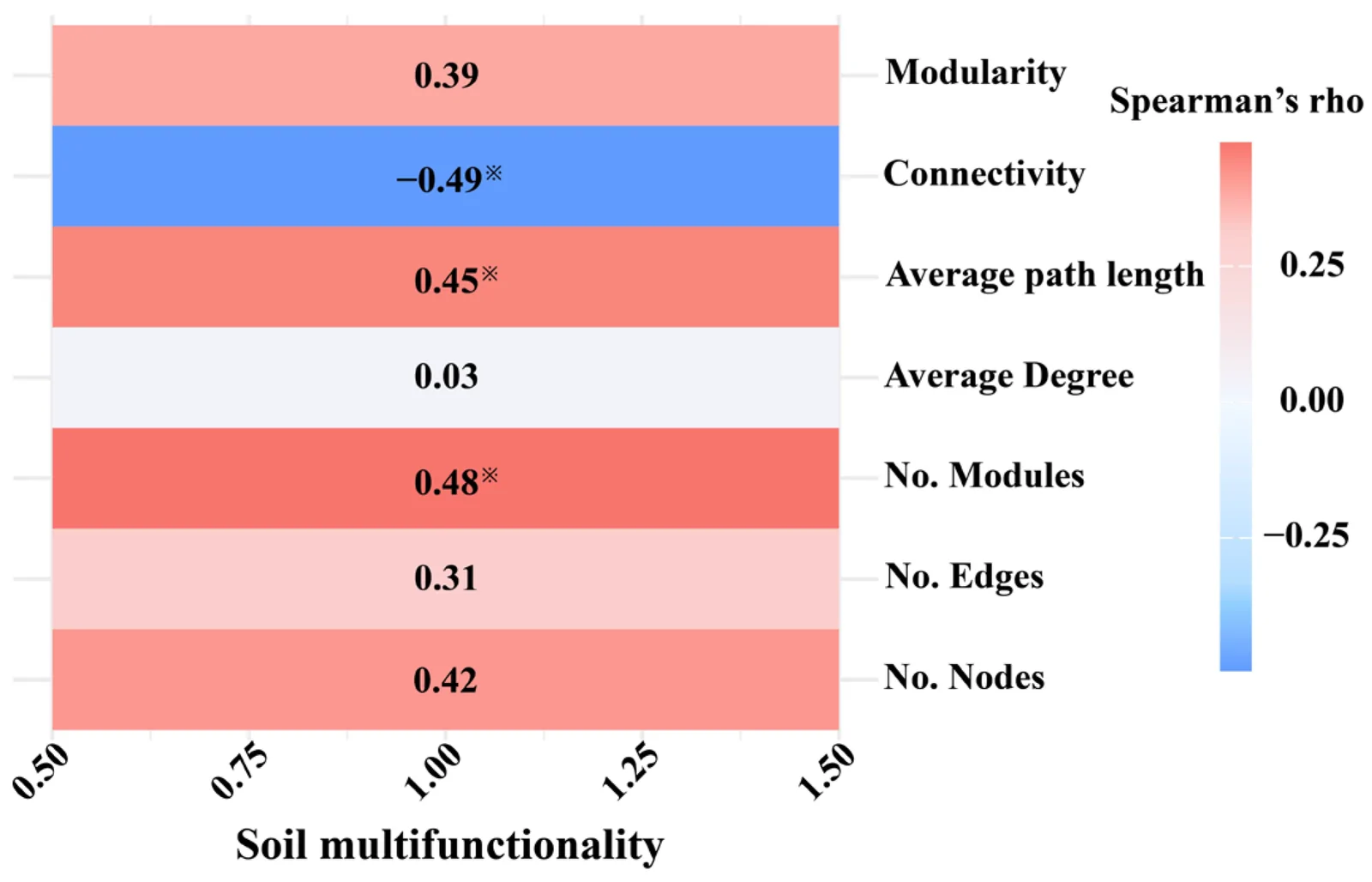
Spearman’s correlation coefficients (ρ) between soil multifunctionality and topological properties of bacteria–fungi inter-domain co-occurrence networks (false discovery rate (FDR) adjusted significance * *p* < 0.05).

No significant relationships were detected between soil multifunctionality and either bacterial or fungal richness or phylogenetic diversity (Figure 4). Among the topological properties of the inter-domain co-occurrence networks, the number of modules and average path length were significantly and positively correlated with soil multifunctionality, whereas connectivity was negatively correlated (Figure 5). However, similar to alpha diversity patterns, key topological network properties did not differ significantly among treatments (Figure S3b).

**Figure 6:**
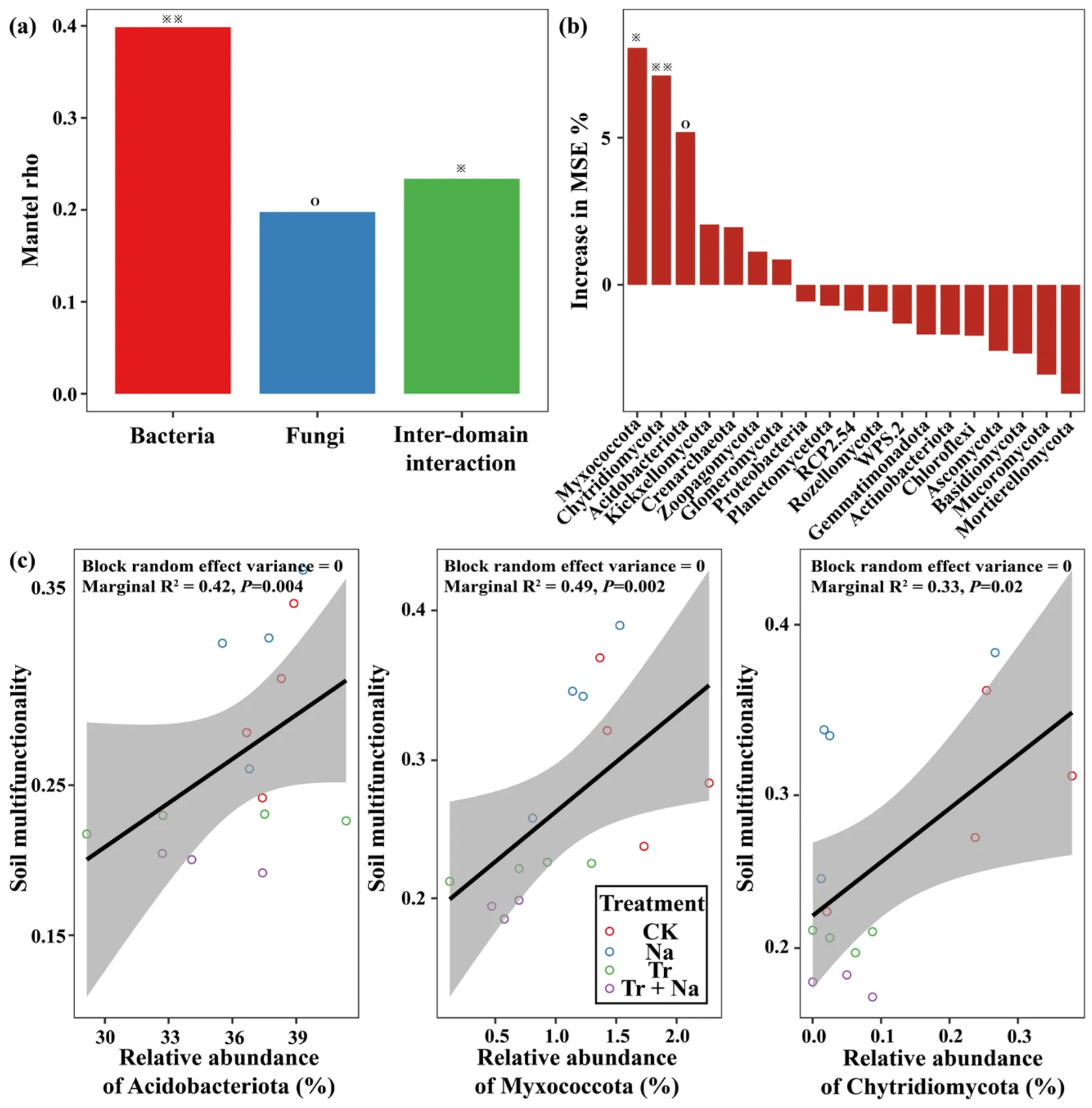
(**a**) Mantel test results showing correlations between soil multifunctionality and bacterial and fungal β-diversity (Bray–Curtis dissimilarity), as well as network topological properties. (**b**) Random forest analysis showing the relative importance of dominant bacterial and fungal phyla in predicting soil multifunctionality. (**c**) Scatterplots showing positive relationships between soil multifunctionality and the relative importance of Acidobacteriota, Myxococcota, and Chytridiomycota, identified as key biomarkers by random forest analysis. Significance levels: ° *p* < 0.1, * *p* < 0.05, ** *p* < 0.01. Lines represent linear mixed-effects model fits with Block as a random intercept; shaded areas indicate 95% confidence intervals. None of the relationships were significant (all *p* > 0.05). Treatment abbreviations: CK, control (no nitrogen addition and no throughfall reduction treatment), Tr, throughfall reduction (covering about 50% area for throughfall exclusion), Na, nitrogen addition (10 g nitrogen m^−2^ yr^−1^), and Tr + Na, combined throughfall reduction and nitrogen addition (covering about 50% area for throughfall exclusion plus 10 g nitrogen m^−2^ yr^−1^).

Mantel tests revealed the strongest correlation between soil multifunctionality and the β-diversity of soil bacterial communities, followed by the topological attributes of the cross-domain microbial network and soil fungal communities (Figure 6a). Random forest analysis (model R^2^ = 0.41, OBB R^2^ = 0.45, *p* < 0.001) further identified bacterial Myxococcota and Acidobacteriota, and fungal Chytridiomycota, as the most important predictors of multifunctionality, as indicated by relatively high increases in mean squared error (MSE) values and significant or marginally significant *p*-values (Figure 6b). Moreover, the relative abundances of these taxa were positively and significantly (or marginally significantly) associated with soil multifunctionality (Figure 6c).

## 4. Discussion

### 4.1. Throughfall Reduction Is the Primary Factor of Reducing Soil Multifunctionality in a Moso Bamboo Forest

Despite receiving relatively high annual precipitation (>800 mm), reduced precipitation and worsening N deposition threaten the functions and survival of moso bamboo forests in the Sichuan Basin [26,27]. Similar to previous studies reporting the negative effects of throughfall reduction on soil multifunctionality [10,23,24], we found that such inhibition becomes even worse when N addition is applied simultaneously. This may not be surprising, because the metabolism of soil microbes requires both a suitable temperature and adequate soil moisture. In subtropical regions, conditions during the growing season generally satisfy temperature requirements; however, throughfall reduction can directly reduce the soil water content and thus inhibit soil microbial functions [46].

In contrast to previous studies reporting negative effects of N addition alone on soil function in moso bamboo forest [22,47], this study did not observe a significant relationship with the independent application of N addition and soil multifunctionality. This discrepancy may be attributed to the condition of N content in local soil. In ecosystems with a low soil N content, N addition often promotes microbial functions relating to biogeochemical cycling [20,48], whereas in N-rich soils, such as those in our research area [28], the positive effects may become negligible. Thus, the joint stress of throughfall reduction and N addition significantly lowers soil multifunctionality. Our findings support the first hypothesis: throughfall reduction significantly decreased soil multifunctionality, and its combination with N addition resulted in the lowest multifunctionality, whereas N addition alone had only minor effects. This can further be proved by our previous manipulation experiment showing that the combination of throughfall reduction and N addition exerted larger declines in ecosystem functions (soil respiration or litter decomposition) than either factor alone [28,49].

Our study was based on a three-year field manipulation experiment, which should be considered a relatively short-term investigation. In addition, soil samples were collected at a single time point rather than across multiple seasons. Consequently, our study may not fully capture seasonal variation in the effects of throughfall reduction and nitrogen addition on ecosystem functioning. Previous studies have also demonstrated that short-term and long-term nitrogen addition can exert distinct effects on soil microbial community structure and ecosystem functioning [50,51]. Therefore, further long-term monitoring is needed to evaluate the responses of soil microbial communities and ecosystem functioning to prolonged throughfall reduction and nitrogen addition. Moreover, multi-seasonal sampling will be essential to better understand how the effects of throughfall reduction and nitrogen addition on ecosystem functioning vary across seasons.

### 4.2. Soil Microbial Diversity Is Less Important than Community Composition in Influencing Soil Multifunctionality Under Throughfall Reduction and N Addition

In line with the second hypothesis, both bacterial and fungal α-diversity remained largely unchanged under the treatments. Microbial diversity is widely recognized as a key contributor to ecosystem functioning across forest and grassland ecosystems [1,8,19,52]. However, in this study, microbial community composition and network structure, rather than taxonomic diversity per se, played a more dominant role in regulating soil multifunctionality (Figure 4, Figure 5 and Figure 6). This finding is consistent with previous observations [8], suggesting that soil multifunctionality is more closely associated with the identity and functional traits of key taxa (e.g., keystone decomposers or nutrient-cycling specialists) and their ecological interactions, rather than with overall species richness [53]. Under an altered soil water content and nutrient regimes (Figure 2 and Figure S4), the reorganization of microbial functional potential, such as shifts in the abundance of taxa harboring nutrient acquisition genes or changes in co-occurrence network topology, can modify process rates and functional coupling even when α-diversity remains stable. Thus, soil multifunctionality may be governed by functional redundancy and community reassembly within a relatively constant diversity pool, reflecting a decoupling between diversity metrics and ecosystem processes.

We further observed a negative correlation between soil multifunctionality and the connectivity of microbial inter-domain co-occurrence networks, accompanied by a positive relationship with the number of network modules. This pattern is consistent with the hypothesis that more modular microbial communities may support ecosystem multifunctionality by promoting ecological compartmentalization, which has been proposed to facilitate niche differentiation and potentially increase functional complementarity among microbial groups [54]. In contrast, highly connected (densely linked) networks tend to be dominated by a limited set of strongly interacting taxa, which may constrain functional breadth. According to this hypothesis, an increased number of modules promotes the formation of semi-autonomous subcommunities, each maintaining particular biogeochemical roles (e.g., carbon, nitrogen, or phosphorus transformations), thereby enhancing ecosystem multifunctionality and buffering the system against perturbations to any single function [55]. Conversely, greater global connectivity may indicate networks dominated by a few central taxa or pervasive interactions that reduce niche differentiation and functional redundancy, ultimately weakening the community’s ability to deliver multiple functions simultaneously [56]. Nevertheless, our network analysis is based on correlation-derived co-occurrence patterns and therefore identifies potential ecological associations rather than direct microbial interactions or functional partitioning. Moreover, because the network was constructed by pooling samples across treatments, treatment-specific rewiring of microbial interactions could not be evaluated. Additionally, we note an apparent tension in our results: network topological properties (connectivity, average path length, number of modules) were significantly correlated with SMF across plots, yet did not differ significantly among treatments. Two factors may explain this pattern. First, the statistical power for detecting treatment effects was limited by replication (*n* = 4 per treatment) relative to the correlation analysis (*n* = 16), and the observed visual separation among treatments in boxplots may reflect real but modest treatment effects that our design could not resolve. Second, network topology may covary with SMF along gradients of within-treatment variation (e.g., microhabitat heterogeneity, stochastic assembly) that are independent of the experimental treatments. While these correlations suggest that microbial co-occurrence architecture may be functionally relevant, they do not provide direct evidence that treatment-induced changes in network organization causally influence soil multifunctionality. Accordingly, future studies combining greater within-treatment replication with experimental manipulation of microbial network structure and trait-based or functional analyses will be valuable for directly testing this mechanistic hypothesis.

Furthermore, Myxococcota, Acidobacteriota, and Chytridiomycota were identified as the key biomarkers contributing to enhanced soil multifunctionality under throughfall reduction and nitrogen addition in our study (Figure 6), which was consistent with the third hypothesis, that the relative abundances of key functional taxa, other than soil microbial alpha diversity, would be the primary driver of multifunctionality. Myxococcota, which was recently reclassified from Deltaproteobacteria into a distinct phylum based on phylogenomic evidence and unique metabolic traits, is highly associated with cellulose degradation [57,58]. In addition, Acidobacteriota and Chytridiomycota are ubiquitous soil taxa that play important roles in polymer degradation and nutrient cycling [59,60]. Although other taxa may maintain specific soil functions through functional redundancy, a reduction in the relative abundance of these key biomarkers could lead to an overall decline in soil multifunctionality.

## 5. Conclusions

By investigating soil multifunctionality, microbial diversity, and cross-domain microbial networks under throughfall reduction and nitrogen addition in a subtropical moso bamboo forest, our study clarifies how multiple global change drivers regulate belowground biodiversity and ecosystem functioning. The results reveal that throughfall reduction significantly suppressed soil multifunctionality, while nitrogen addition exerted negligible impacts on this function. Co-occurrence of the two treatments further aggravated the decline in soil multifunctionality. Such functional variation was primarily driven by structural reorganization of soil microbial communities, rather than shifts in microbial α-diversity. It is important to recognize that soils in moso bamboo forests exhibit substantial spatial heterogeneity in physicochemical properties. Therefore, the patterns observed here may reflect site-specific processes rather than universally applicable mechanisms. Future studies across diverse soil types and climatic regions are needed to validate the generality of these findings and to further elucidate the microbial pathways linking multiple global change drivers to ecosystem multifunctionality.

## Data Availability

The original contributions presented in this study are included in the article/Supplementary Materials. Further inquiries can be directed to the corresponding author.

## Supplementary Materials

The following supporting information can be downloaded at https://www.mdpi.com/article/10.3390/microorganisms14081619/s1, Figure S1. Cluster analysis of soil functions. Determination of the optimal number of clusters (k) for grouping ecosystem functions in the multifunctionality index using (a) Elbow method based on within-cluster sum of squares (WSS), and (b) average silhouette width across k = 2 to 8. (c) Dendrograms showing main clusters of soil functions for soil multifunctionality. PPO, Polyphenol oxidase; PHE, Phenoloxidase peroxidase; ACP, Acid phosphatase; AP, total available phosphorus; TN, total nitrogen; TP, total phosphorus; BX, β-Xylosidase; CB, Cellobiohydrolase; NAG, β-N-acetyl-glucosaminidase; NO_3_^−^-N, Nitrate nitrogen; AG, α-Glucosidase; NH_4_^+^-N, Ammonium nitrogen; BG, β-Glucosidase; LAP, Leucine Aminopeptidase; SOC, Soil organic carbon. High values of these parameters indicate higher nutrient availability, resource accumulation or microbial mediated carbon, nitrogen, phosphorus processes; Figure S2. Out-of-bag (OOB) error stabilization for the random forest model predicting multifunctionality from phylum-level relative abundances. The grey line shows raw OOB MSE per tree; the dark line shows a 50-tree rolling mean. The red dashed line indicates the final OOB MSE; the blue dotted line marks the tree at which the smoothed error stabilized within 5% of the final value; Figure S3. Inter-domain co-occurrence networks of soil bacteria and fungi with nodes colored by bacteria and fungi (a). Boxplots showing differences in the topological properties of microbial inter-domain co-occurrence networks among treatments (b). No significant differences (Friedman tests with block as the blocking factor, χ^2^ = 6.3, 3.9 and 3.9, respectively, *p* > 0.05) of the topological properties were observed among treatments, as shown by the same lowercase letter; Figure S4. Scatterplots showing relationships between soil multifunctionality and soil moisture content (a) and pH (b). Shaded areas indicate 95% confidence intervals.
