# Supplementary material for: Throughfall Reduction Other than Nitrogen Addition Threatens Soil Multifunctionality in a Subtropical Moso Bamboo Forest
Source: Microorganisms. 2026 Jul 24;14(8):1619. doi: 10.3390/microorganisms14081619 (PMC13515568; doi:10.3390/microorganisms14081619)
Supplement: Supplementary file 1 [file microorganisms-14-01619-s001.zip › microorganisms-4376747-supplementary.pdf]

Electronic Supporting Information for

Throughfall reduction other than nitrogen addition threatens soil multifunctionality in a subtropical moso bamboo forest

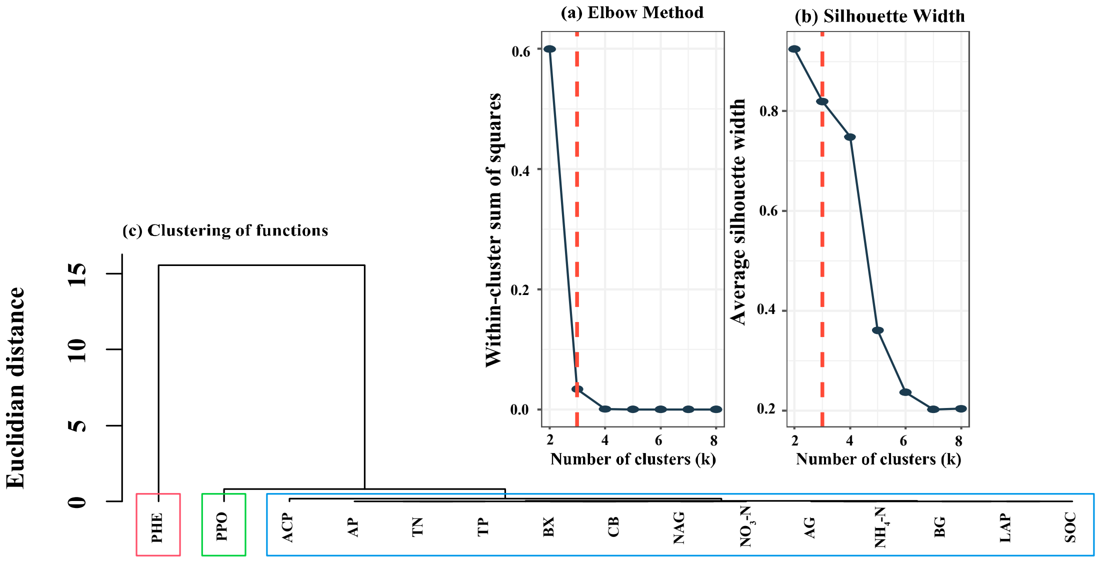

Figure S1 Cluster analysis of soil functions. Determination of the optimal number of clusters (k) for grouping ecosystem functions in the multifunctionality index using (a) Elbow method based on within-cluster sum of squares (WSS), and (b) average silhouette width across k = 2 to 8. (c) Dendrograms showing main clusters of soil functions for soil multifunctionality. PPO, Polyphenol oxidase; PHE, Phenoloxidase peroxidase; ACP, Acid phosphatase; AP, total available phosphorus; TN, total nitrogen; TP, total phosphorus; BX, β-Xylosidase; CB, Cellobiohydrolase; NAG, β-N-acetyl-glucosaminidase; NO_3^--N, Nitrate nitrogen; AG, α-Glucosidase; NH_4^+-N, Ammonium nitrogen; BG, β-Glucosidase; LAP, Leucine Aminopeptidase; SOC, Soil organic carbon. High values of these parameters indicate higher nutrient availability, resource accumulation or microbial mediated carbon, nitrogen, phosphorus processes.

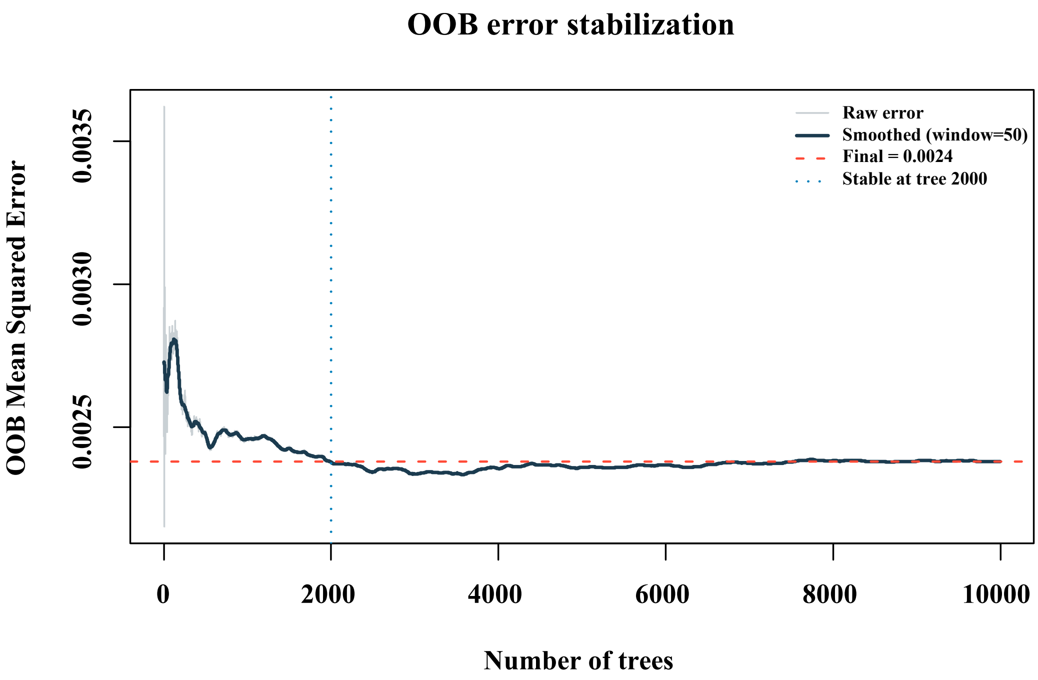

Figure S2. Out-of-bag (OOB) error stabilization for the random forest model predicting multifunctionality from phylum-level relative abundances. The grey line shows raw OOB MSE per tree; the dark line shows a 50-tree rolling mean. The red dashed line indicates the final OOB MSE; the blue dotted line marks the tree at which the smoothed error stabilized within 5% of the final value.

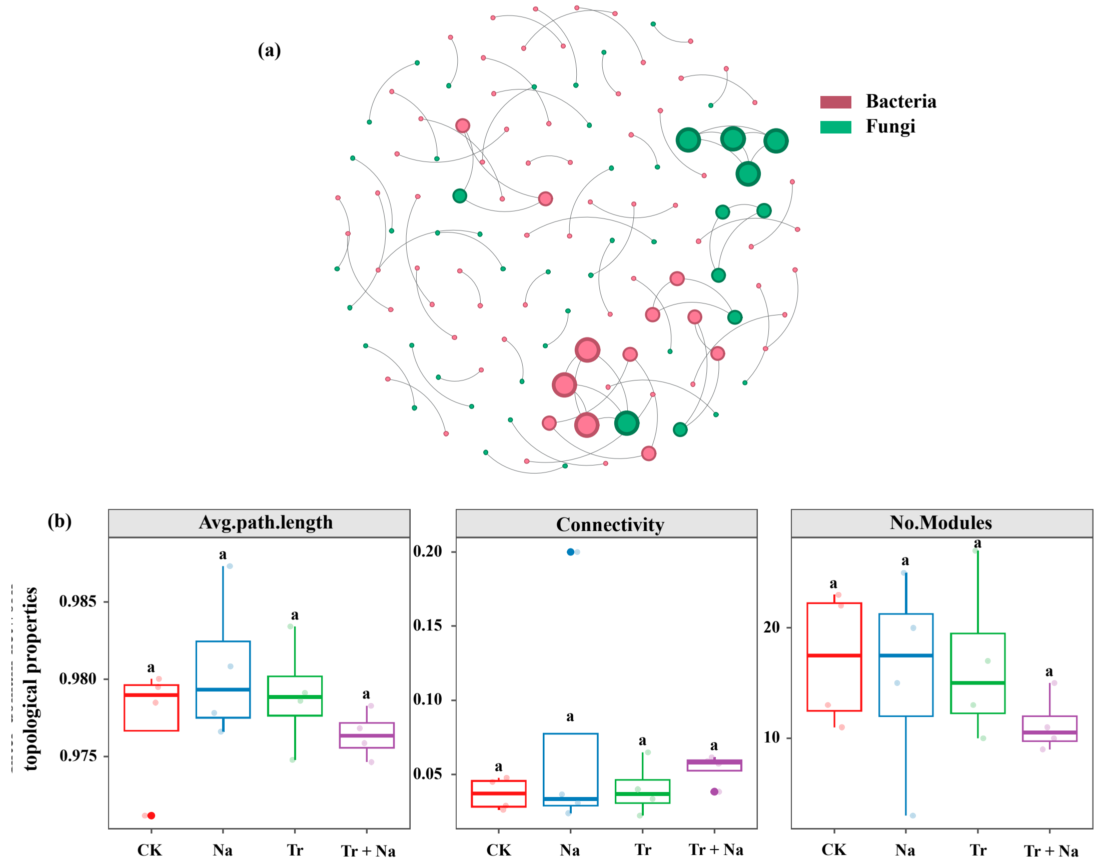

Figure S3 Inter-domain co-occurrence networks of soil bacteria and fungi with nodes colored by bacteria and fungi (a). Boxplots showing differences in the topological properties of microbial inter-domain co-occurrence networks among treatments (b). No significant differences (Friedman tests with block as the blocking factor, $\chi^2 = 6.3$, 3.9 and 3.9, respectively, $P > 0.05$) of the topological properties were observed among treatments, as shown by the same lowercase letter.

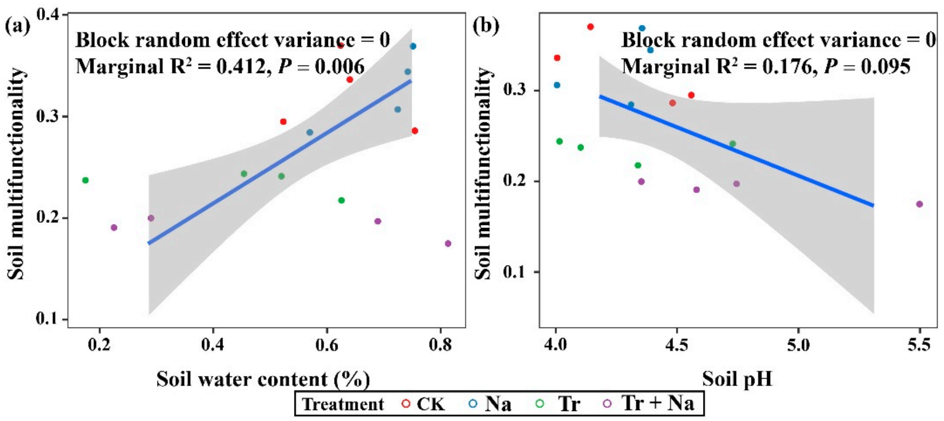

Figure S4 Scatterplots showing relationships between soil multifunctionality and soil moisture content (a) and pH (b). Shaded areas indicate 95% confidence intervals.
